# Redaporfin induces immunogenic cell death by selective destruction of the endoplasmic reticulum and the Golgi apparatus

**DOI:** 10.18632/oncotarget.25798

**Published:** 2018-07-27

**Authors:** Lígia Catarina Gomes-da-Silva, Liwei Zhao, Luis G. Arnaut, Guido Kroemer, Oliver Kepp

**Affiliations:** Faculty of Medicine, University of Paris Sud, Kremlin-Bicêtre, France; Metabolomics and Cell Biology Platforms, Gustave Roussy Cancer Campus, Villejuif, France; Institut National de la Santé et de la Recherche Médicale UMR1138, Equipe 11 labellisée Ligue Nationale contre le Cancer, Centre de Recherche des Cordeliers, Paris, France; Université Paris Descartes, Sorbonne Paris Cité, Paris, France; Pôle de Biologie, Hôpital Européen Georges Pompidou, APsupp-HP, Paris, France; Metabolomics and Cell Biology Platforms, Gustave Roussy Cancer Campus, Villejuif, France; Institut National de la Santé et de la Recherche Médicale UMR1138, Equipe 11 labellisée Ligue Nationale contre le Cancer, Centre de Recherche des Cordeliers, Paris, France; Université Paris Descartes, Sorbonne Paris Cité, Paris, France

**Keywords:** photodynamic therapy, redaporfin, immunogenic cell death, ER stress, Golgi damage

## COMMENTARY

Redaporfin, a synthetic bacteriochlorin, is an experimental anticancer agent, which is currently being evaluated in clinical trials that explore the efficacy of photodynamic therapy (PDT) against advanced head and neck cancer (NCT02070432). Similar to other photosensitizing agents, redaporfin exerts its cytotoxic potential exclusively upon the absorption of photons, i.e. after administration of light with the appropriate wavelength. Typically, systemic administration of the photosensitizer is followed by local illumination of the tumor with the aim of inducing site-specific cytotoxicity. The photoactivation of redaporfin triggers the generation of reactive oxygen species (ROS) that in turn leads to the spatially restricted damage of cellular structures and subsequent cellular demise. As compared to other PDT agents, redaporfin is endowed with an elevated ROS quantum yield, high stability, which facilitates its redistribution into cellular, or subcellular compartments and an absorption spectrum that allows for the treatment of relatively deep lesions [1]. As compared to standard chemotherapy, which exposes the whole organism to treatment related cytotoxicity, PDT allows to spatially limit toxicity and therefore reduces overall side effects, while locally destroying tumor tissue and vasculature.

The anticancer efficacy of PDT in general and redaporfin in particular has been ascribed to the elicitation of antitumor immunity [2], because PDT is more efficient in immunocompetent than in immunodeficient mice. In preclinical studies, redaporfin achieved 85 % cure in immunocompetent mice bearing syngeneic transplanted tumors, abscopal effects on distant lesions and generated immunological memory that protected animals against rechallenge with live tumor cells of the same type [3-5]. The antitumor immunity of PDT has been ascribed to the induction of immunogenic cell death (ICD) with traits of endoplasmic reticulum ER-stress and the emission of danger associated molecular pattern molecules (DAMPs), such as the cell surface exposure of calreticulin (CALR) and the extracellular release of adenosine triphosphate (ATP) and high mobility group box 1 (HMGB1) protein [6, 7].

**Figure 1:**
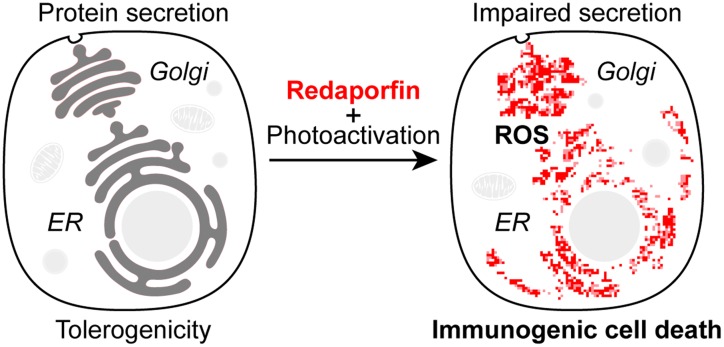
Redaporfin-induced cellular stress and compartmental alteration (A) Redaporfin accumulates in the endoplasmic reticulum (ER) and Golgi apparatus (GA). Photoactivation of redaporfin triggers the generation of reactive oxygen species (ROS), which in turn leads to local damage, dysfunction of the ER/GA network and impairment of global protein secretion. In addition, redaporfin-induced ER/GA damage operates upstream of mitochondria to induce apoptosis. *In vivo* the photodynamic therapy with redaporfin triggers cell death of the primary tumor and elicits antitumor immunity that is able to control distant leasions.

Recently, we have characterized the cytotoxic mode of action of redaporfin and showed that redaporfin has a selective tropism for the endoplasmic reticulum (ER) and Golgi apparatus (GA). Thus, redaporfin-mediated photodynamic therapy selectively inflicts ROS-dependent damage to the ER/GA compartments (Figure 1) [8]. The redaporfin PDT-induced damage of ER/GA sets of a hierarchically organized cascade of signaling events involving other organelles including mitochondria to stimulate classical apoptosis. Of note, genetic and pharmacological interventions that inhibit mitochondrial membrane permeabilization reduced the short-term cytotoxicity of redaporfin PDT, yet failed to protect cells from PDT-induced killing in clonogenic assays. This points toward redundant mechanism that would safeguard cellular self-destruction in response to the irreversible impairment of vital organelles.

Redaporfin PDT leads to the induction of ER stress while compromising canonical, GA-dependent protein secretion. Accordingly, the transport of model secretory cargos monitored by the retention using selective hooks (RUSH) assay was compromised and global cytokine secretion was blocked. The emission of DAMPs in response to redaporfin PDT occurred despite the complete and irreversible inactivation of GA-dependent protein secretion, in accord with the idea that ATP and HMGB1 release as well as CALR exposure occur through GA-independent mechanisms. It has been suggested that the surface exposure of CALR depends on the eukaryotic translation initiation factor 2-alpha (eIF2α) kinase 3 (EIF2AK3)-dependent structural reorganization of the ER that facilitates the formation of ER and plasma membrane contact sites and thus operates independently of classical protein trafficking [9]. However, in the case of redaporfin PDT, the crucial kinase responsible for eIF2α phosphorylation is another one, namely EIF2AK1 (rather than EIF2AK3), pointing to additional mechanisms [8] that require further mechanistic exploration.

We have previously shown that the peptide LTX-401 selectively targets GA to induce ICD. At difference with redaporfin PDT, LTX-401 induces a reversible and ROS-independent inhibition of conventional protein secretion [8]. However, both agents strongly perturb the GA microanatomy, and mitochondrial permeabilization setting of the apoptotic cascade can be delayed by GA-dissipating drugs (such as brefeldin A and golgicide), deferring cell death induced by both redaporfin PDT and LTX-401. These results suggest that chemically unrelated compounds targeting the GA through distinct mechanisms can trigger ICD.
